# Beliefs about the causes and treatment of common mental illnesses and suicidality in rural Uganda

**DOI:** 10.1192/bjo.2025.10066

**Published:** 2025-07-08

**Authors:** Yang Jae Lee, Rita Mbabazi, Ondine Jevremov, Shakira Nakaweesi, Ella Brodey, Jason Wykoff, Roshan Sivakumar, Rauben Kazungu, Ibrahim Ssekalo, Robert Rosenheck, Alexander C. Tsai

**Affiliations:** Department of Psychiatry, Yale University, New Haven, Connecticut, USA; Empower Through Health, Iganga, Uganda; Williams College, Williamsburg, Massachusetts, USA; Cavendish University, Kampala, Uganda; Washington University in St. Louis, Missouri, USA; Institute for Social and Preventive Medicine, University of Bern, Switzerland; Center for Global Health and Mongan Institute, Massachusetts General Hospital, Boston, Massachusetts, USA; Harvard Medical School, Cambridge, Massachusetts, USA; Mbarara University of Science and Technology, Uganda

**Keywords:** Global mental health, Uganda, stigma

## Abstract

**Background:**

Mental illnesses constitute a large and escalating portion of the global burden of disease, particularly in low- and middle-income countries like Uganda. Understanding community perceptions towards mental illness is crucial for developing effective interventions.

**Aims:**

To explore beliefs about the perceived causes and treatment of common mental illnesses (depression, anxiety, alcohol use disorder) and suicidality in rural eastern Uganda.

**Method:**

Qualitative study using 31 in-depth interviews and 4 focus group discussions with healthcare workers, community health workers, community leaders and general community members in Buyende District, Uganda. Vignettes were used to depict mental illnesses to elicit perceptions, and data were analysed using the framework method.

**Results:**

Two main themes emerged: perceived causes and treatment of mental illness. Participants identified three primary perceived causes: psychosocial (predominantly financial stress), biological and supernatural. Community support was most frequently endorsed as a perceived effective treatment, followed by biomedical interventions and alternative therapies.

**Conclusions:**

This study identifies common beliefs regarding the causes and perceptions of mental illness in rural Uganda. The predominant focus on financial stressors as a cause of mental illness, coupled with strong emphasis and belief in the effectiveness of community-based support as treatment, highlights the need for context-specific mental health interventions.

Mental illnesses constitute a large and escalating portion of the global burden of disease. Between 1990 and 2019, the proportion of disability-adjusted life years (DALYs) lost annually to mental illnesses rose from 3.1 to 4.9% of overall disease burden.^1^ This burgeoning concern is particularly pronounced in low- and middle-income countries (LMICs), which comprise 80% of the mental health global disease burden.^2^ Despite the severity of the issue, mental health services in LMICs remain underfunded, exacerbating the challenges faced by individuals grappling with mental illnesses.^2^ In Uganda, a low-income country, only 15% of those with mental illness access any formalised mental health treatment, and less than 1% of the nation’s healthcare budget is allocated to mental health services.^3^ The repercussions are significant, leading to a critical shortage of trained specialists and hindering the widespread implementation of evidence-based treatments.^3,4^

Recognising the interconnectedness of mental health and community dynamics, the prognosis of mental illness is intricately linked to community-level factors.^5^ Community attitudes towards mental illness can play a pivotal role in either fostering mental health awareness or perpetuating stigma and discrimination.^6,7^ Therefore, to meaningfully address mental health needs in LMICs, it becomes imperative to comprehend how both individuals and key community stakeholders, such as healthcare workers and local leaders, within these communities conceptualise mental illness. Interventions designed to enhance mental health outcomes must be grounded in an understanding of existing community perceptions.^8^ The vital elements of community knowledge, including perceived causes, effective treatment options and help-seeking behaviours, are essential in crafting strategies to adequately address mental illness.^9,10^

An intriguing facet of mental healthcare seeking in LMICs lies in the diverse modalities individuals pursue in the absence of robust biomedical mental healthcare systems.^11,12^ Traditional and faith healers (TFHs) play an important role, particularly in rural areas of LMICs, offering accessible and culturally relevant care that incorporates holistic support.^13,14^ This underscores the importance of understanding and integrating alternate modalities of mental healthcare in Africa.

Prior investigations have uncovered relatively poor community knowledge regarding causation and pervasive stigma towards mental illness.^15,16^ Discrepancies in beliefs about perceived causes of mental illness and stigmatising attitudes within Africa highlight the need for context-specific exploration.^17,18^ In light of this, our qualitative exploratory study aims to elucidate the beliefs of healthcare workers, community health workers, community leaders and general community members in rural eastern Uganda regarding common mental illnesses (depression, anxiety and alcohol use disorder) and suicidality. Through in-depth interviews (IDIs) and focus group discussions (FGDs), participants engaged with vignettes describing characters with these mental health conditions, allowing us to probe their views on perceived causes and treatment. By gaining insights into community beliefs, our study aspires to inform the development of culturally appropriate mental health interventions that align with community perspectives in rural Uganda.

## Method

### Study setting

We conducted this study in Buyende District, a rural district in eastern Uganda with an approximate population of 400 000.^19^ Buyende District is located roughly 150 km from the capital city, Kampala. Specifically, the sub-counties Irundu (approximately 26 000 inhabitants) and Kagulu (approximately 58 000 inhabitants) were the focus of this study. Buyende District has poor infrastructure and limited resources, especially in health, with no hospitals; 1 health centre IV (designed to provide curative, preventive, maternity, out-patient and in-patient services and supervise health centres III and II with a target catchment area of 100 000 people); 7 health centres III (designed to provide basic preventive, promotive and curative care, with a target catchment area of 20 000 people); and 14 health centres II (designed to provide out-patient care, including antenatal and preventive care, with a target catchment area of 10,000 people).^20^ Most residents are subsistence farmers, and food and water insecurity are common.^19,21^

### Study design and data collection

This study employed a qualitative design using the framework method to understand how key stakeholders conceptualise and understand mental illness in their context.^22^ The study was explained to the local leadership and community health workers in the study areas, and they helped identify key informants. Participants were selected through convenience sampling, but we ensured that there was sufficient variety in age, gender, background and location. All participants were over 18 years old.

Following identification, trained research assistants fluent in Lusoga approached participants in the field to obtain verbal and written informed consent using a standardised form, translated into Lusoga and back-translated for fidelity. If participants could not read or write, a fingerprint was used alongside a witness signature. Consistent with local etiquette, there was a small monetary incentive given to each respondent for their participation. The study incentive was not disclosed to community leaders during the initial participant identification process. The offer and amount of study incentive were discussed only during the informed consent process, after participants had agreed in principle to participate.

We conducted 31 IDIs and 4 FGDs. Eight community health workers (community-selected volunteers who received a basic 5- to 7-day training in health, including child health, common diseases and sexual and reproductive health),^23^ 6 health workers (primary healthcare workers working in local health centres consisting of nurses, midwives and clinical officers), 11 community leaders and 6 community members participated in the IDIs. FGDs consisted of one group of six community health workers, one group of eight community leaders, one group of eight health workers and one group of eight general community members. In each FGD, we assigned each participant a number they utilised to identify themselves when speaking. No participants were excluded. All health workers had received at least one year of schooling (certificate minimum) past secondary school. All community leaders were on the government registries of elected leaders. All community health workers were official village health team (VHT) members appointed by their village councils.

All interviews were conducted in English or Lusoga, based on participant preference, and took place in locations convenient to them – most often in homes or workplaces. Interviewers were Ugandan research assistants with a background in social science or public health, all of whom received training in qualitative methods and research ethics. US research assistants observed and supported audio recording but did not participate in interviewing.

Participants were first asked to describe any mental illnesses with which they were familiar and what distinguished different mental illnesses from each other. They were then read a vignette illustrating the experience of a man or a woman with depression and were asked: (a) Would you call this a mental illness? Why or why not? (b) What do you think causes this condition? And (c) What would help this person? While the responses were open-ended and allowed participants to provide diverse perspectives, the overarching themes of causes and treatment were directed in part by the questions posed during the interviews. The procedure was then repeated for vignettes illustrating anxiety and alcohol use disorder. Similar questions were then asked regarding participants’ ideas on suicide. Research assistants asked probing questions, and participants provided free-form answers.

The IDIs and FGDs, each lasting 30–90 min, were audio recorded, translated from Lusoga to English and transcribed. Each FGD was facilitated by either two or three research assistants: one acted as the primary moderator while the others took notes and observed non-verbal communication and group dynamics to ensure comprehensive data collection. Data collection proceeded until content saturation was achieved.

The vignette illustrating depression was adapted from a study conducted in south-western Uganda and modified to fit the local context.^24,25^ Vignettes illustrating anxiety and alcohol use disorder were developed through a collaborative effort from Ugandan and US researchers, including a Ugandan psychiatric clinical officer who reviewed them to ensure that they resonated with the lived experiences of participants in the study setting.

### Data analysis

Interviews conducted in Lusoga were translated into English by a native Lusoga speaker and transcribed. Interviews conducted in English were transcribed directly. We used the framework method to organise recurring themes in the data.^22^ The first three IDI transcriptions were coded individually, then the study team members met to compare the codes and agreed on which ones to apply to all subsequent transcripts. Study team groups then coded the remaining transcripts separately.

To ensure rigour, we triangulated data by comparing findings between the IDIs and FGDs using multiple coders with different cultural perspectives, and then by verifying interpretations through team discussions. Each interview transcript was reviewed by either two or three study team members who familiarised themselves with the content and applied a paraphrase, or code, to passages that they interpreted as important. Study team members coded the first three to five transcripts of each respondent group separately (in groups of two or three). Each group consisted of at least one Ugandan and one US study author. When there were questions raised about cultural or linguistic interpretation during coding, the team consulted with the Ugandan researchers and original interviewers to ensure accurate understanding of participants’ meanings in their cultural context. The study team members then met to compare the codes and agreed on codes to apply to all subsequent transcripts. Study team groups then coded the remaining transcripts separately.

We organised our findings by grouping related codes into distinct categories, which formed the basis of our analytical structure. This structure was continuously refined as we analysed additional transcripts, incorporating new codes as they emerged. The final step involved transforming our analytical framework into a hierarchy of themes and sub-themes. This process required a comprehensive review of our established categories, identifying underlying patterns and concepts that linked multiple categories. These overarching ideas were then developed into main themes. Within each theme, we identified more specific elements or variations, which became our sub-themes. This hierarchical approach allowed us to present our results in a logical and meaningful manner, emphasising the key insights from our data while preserving the subtle complexities uncovered during our analysis.

### Ethical considerations

The authors assert that all procedures contributing to this work comply with the ethical standards of the relevant national and institutional committees on human experimentation, and with the Helsinki Declaration of 1975 as revised in 2013. This research study was approved by the Institutional Review Boards of The AIDS Support Organization, Uganda (no. TASO-2023-222) and Yale University (no. 2000034605). In accordance with national guidelines, we received approval to conduct the study from the Uganda National Council of Science and Technology (no. SS1860ES).

## Results

We organised participant responses using two overarching analytic categories: perceived causes and perceived treatments for mental illness. These categories were shaped in part by the structure of the interview questions, which asked participants about what they believed had caused the condition and what they believed would help. Within each category, inductively derived sub-themes reflected the diversity and nuance of community perspectives (Box 1).

Box 1 Themes identified from the in-depth interviews and focus group discussions

Theme 1: Perceived causes of mental illness

Sub-theme 1: Psychosocial

Sub-theme 2: Biological

Sub-theme 3: Supernatural

Theme 2: Treatment of mental illness

Sub-theme 1: Community care

Sub-theme 2: Biomedical care

Sub-theme 3: Alternative care

### Perceived causes of mental illness

Participants endorsed three major perceived causes of mental illness: psychosocial, biological and supernatural.

#### Psychosocial causes of mental illness

The primary cause of mental illness reported by participants was financial stress. Across all examined disorders, respondents consistently highlighted the important impact of financial concerns on mental health. Participants emphasised that the apprehension related to monetary issues could manifest in worrying or altered behaviour:

‘Poverty has done a great job bringing diseases into peoples’ lives. Because of poverty, people tend to think too much about what to do.’ – IDI, male community member

Familial stress also emerged as another prevalent factor contributing to mental illness among participants. The reported instances encompassed a spectrum of challenges, including descriptions of abuse, neglect or loss. However, our analysis did not reveal a discernible correlation between specific familial stressors and the manifestation of particular disorders:

‘Having a lot of stress and thinking too much. For example, if you have lost your family or if you have a family that doesn’t understand you. You may also have a husband who mistreats you.’ – IDI, female community leader

Furthermore, participants identified emotional states as contributing to the development of mental illness. Specifically, when examining suicidal ideations, participants frequently cited shame or guilt as potential causative factors. Notably, some community members speculated that someone who has committed a crime might have difficulty coping with their circumstances, often leading to a heightened risk of depression or suicidal ideation:

‘It could be because a person has committed a crime and does not have a family to clear their name so they choose to kill themselves.’ – IDI, female community leader

In the context of alcohol use, a distinct factor highlighted by participants was the influence of peer pressure. Respondents consistently pointed out that the societal environment and cultural norms in Uganda tend to foster a culture of drinking, leading to the development of dependency issues:

‘It could be peer influence that tells him that he should go and take some alcohol. They can even buy for him. So when they are buying alcohol for him, he may get scared to stop because these are his friends and they even buy him alcohol so that can cause them to become alcoholics.’ – IDI, male community health worker

Furthermore, cultural norms propagated stigma surrounding mental health disorders. Numerous participants reported instances of discrimination and judgement directed towards individuals unable to control their emotions, with heightened prevalence in cases of alcohol use disorder and suicidal ideation. This cultural stigma is further intensified by legal restrictions, as evidenced by capital punishment for suicide attempts within the Uganda legal system:

‘They would judge him because he has not been able to bear the situation because if he had hold of the situation he would not have committed^26^ suicide. This person is used as an example in society for people to bear their situations and they shouldn’t think about killing themselves, either by hanging or taking poison.’ – IDI, male community leader

In summary, participants consistently identified psychosocial stress, including financial, familial, cultural and emotional, as a major cause of mental illness. While there are various attitudes toward the psychosocial causes of poor mental health, community standards and stigma in Uganda may also play a role.

#### Biological causes of mental illness

Participants commonly attributed mental illness to biological factors, often referring to them as diseases of the brain. The prevailing perception was that these biological factors had a discernible impact on attitudes and behaviour:

‘I think it’s a disease like any other normal disease we get. It’s in your brain which makes someone get a mental illness’ – IDI, male community health worker

Among the reported beliefs within the studied population, mental disorders such as depression and anxiety were often linked to other diseases such as malaria or HIV. Participants sometimes used the term ‘crazy’ to describe a general descriptor for altered mental states, which could encompass various conditions ranging from common mental illnesses such as psychosis:

‘Malaria can also cause someone’s brain to go crazy.’ – IDI, male community leader

Healthcare workers, in particular, associated suicide with depression, emphasising the presence of these conditions as comorbidities:

‘She has taken time over worrying, what comes of that? Depression. And depression leads to what? Death.’ – IDI, male healthcare worker

In conclusion, participants commonly associated mental illness with biological factors, framing it as a disease of the brain with discernible impacts on attitudes and behaviour. Participants also discussed links between mental illnesses and other medical conditions.

#### Supernatural causes of mental illness

The influence of witchcraft on one’s mental health status was controversial, with only a minority of participants acknowledging it as a potential cause. Most participants who endorsed supernatural causes of mental illnesses were general community members:

‘Maybe it’s witchcraft. A person may be there, confused, not understanding what they’re doing and not doing their usual work because someone bewitched them, to change their lifestyle and their good ways of living.’ – IDI, female community member

While a few respondents still associated witchcraft with certain mental disorders, particularly depression and suicide, a prevailing sentiment emerged among many participants, dismissing this belief as outdated thinking:

‘The problem was about thoughts that caused someone not to think straight and to run mad. So because of this we may think it’s witchcraft but it’s not witchcraft. Yes.’ – male community leader

### Treatment for mental illnesses

A second major theme identified in the data was related to with treatment of mental illnesses. Participants proposed three potential treatment strategies for people with mental illnesses: community care, biomedical interventions and alternative therapeutic modalities.

#### Community care for people with mental illness

Participants frequently believed that community support and counselling were the most effective way of treating mental illnesses. Participants highlighted active listening, facilitating connections to community events and material assistance as being helpful to those with mental illness:

‘I would talk to the person and encourage them to help them get out of their depressed state. You need to encourage such a person if possible. You can even give them some foodstuffs to encourage them to get back to their normal self.’ – IDI, male community leader

Some participants shared that they would encourage someone with a mental illness to accept their situation and not worry about things outside of their control:

‘You need to talk to the person and tell them to stop being worried, and to bear with the situation they are going through and do whatever you can. What you can’t do, what is out of your power, you leave it.’ – IDI, male community leader

Participants consistently expressed a belief in the efficacy of community support and counselling as vital components of mental illness treatment.

#### Biomedical care for people with mental illness

Some participants expressed faith in biomedical care; a considerable number of individuals advocated for hospitals and health centres to be recognised as the primary sources of comprehensive care:

‘They can also take her to the hospital where the doctors can give her some help and tell her exactly what is wrong with her so that she can get out of that situation.’ – IDI, male community leader

Additionally, health workers recommended the use of medications, including antidepressant medication and/or medication, to mitigate alcohol cravings:

‘I would also encourage this person to seek medical treatment. For example, we have antidepressant tabs they can prescribe for this person in order to overcome depression’ – FGD, health worker

Participants shared an understanding that biomedical care can be helpful for treating mental illness.

#### Alternative care for people with mental illness

Alternative methods of mental illness treatment were less frequently endorsed. They were primarily mentioned for the treatment of depression and anxiety, with herbal remedies emerging as one of the notable alternatives discussed by participants:

‘If such a person approaches me, I tell them that there is Basoga herbal medicine. It helps you not worry too much or do a bad thing to yourself.’ – IDI, female community leader

Some participants suggested the utilisation of religious healers, emphasising the importance of aligning such interventions with the patient’s belief system:

‘I would empower him to seek spiritual guidance from church leaders, these people who understand these church leaders. Or if he is Muslim, still refer that colleague to those Muslim heads.’ – FGD, healthcare worker

Individuals actively sought avenues to assist themselves or support community members in coping with mental illness. However, many participants stated that there was limited availability of nearby facilities and affordable resources. A notable consensus among participants was the call for government intervention to facilitate access to care and address the societal stigma associated with discussing and treating mental disorders:

‘Africans are difficult people because most of them don’t want to go to hospitals to be told things about their lifestyles so the government should get a way of delivering services to people who take alcohol at their homesteads, for example, counselling about quitting alcohol and giving them medicine because many of them fear going to the hospitals and it may look like stigma.’ – IDI, male community member

In conclusion, alternative methods of mental illness treatment were less frequently endorsed but regarded as potentially helpful.

## Discussion

In this qualitative study of multiple stakeholders in a rural region of eastern Uganda, key informants were asked about their beliefs concerning perceived causes and helpful treatments for three mental illnesses (depression, anxiety and alcohol use disorder) and suicidality. We found that participants endorsed varying causes of mental illness – psychosocial, biological and supernatural – with psychosocial explanations, particularly financial stressors, dominating most narratives. Additionally, participants emphasised the role of community support in helping those with mental illness, while also acknowledging the role of biomedical and alternative care.

While ‘causes’ and ‘treatment’ were analytic categories shaped in part by the structure of the interview prompts, the content within these categories – including the prominence of poverty, belief in biological mechanisms and community-based approaches to care – emerged inductively from participant responses. In discussions regarding the aetiologies of mental illness, the pervasive effect of poverty on mental health was recurrent. Buyende District, the site of the study, is a rural district that is impoverished relative to other areas of Uganda, a low-income country.^19^ The relationship between poverty and poor mental health has been reported in numerous studies.^27,28^ While consumption demonstrates an equivocal relationship with mental wellness,^29^ food insecurity and housing exhibit a consistent and strong association with common mental illnesses.^28,30,31^ In Buyende District, where most individuals are subsistence farmers, food insecurity is prevalent and temporary housing is common.^15^ Given the socioeconomic context of Buyende District, it is unsurprising that financial stressors were rated as a major factor in causation of mental illness by the study participants. The focus on psychosocial causes of mental illness is consistent with global findings,^32^ although not necessarily with findings from other parts of Africa,^16,17^ accentuating the necessity of context-specific exploration. Findings from other pathways to care studies in Uganda further support the importance of understanding community beliefs about mental illness. For severe mental illness such as schizophrenia, supernatural explanations – including witchcraft and spirit possession – are more commonly endorsed, often leading individuals to seek help from traditional or faith healers.^33^

The dominance of community support as a proposed treatment aligns with the prevalent identification of psychosocial stressors as a major aetiological factor for mental illness in Buyende District. Given that community care emphasises a holistic approach, focusing on active listening, material assistance and communal support, it resonates with the community’s understanding of mental health challenges as being deeply rooted in psychosocial dynamics. This is potentially promising for developing community mental healthcare interventions,^34^ which are associated with better outcomes than standard models of mental healthcare.^35^ At the same time, the understanding of mental illness through a biological lens aligned with preferences for biological treatment. Additionally, endorsement of spiritual causes or witchcraft among some participants corresponded to the consideration of alternative methods, such as prayer, for treatment. It is important to note that participants did not view modalities of treatment as mutually exclusive, which is supported by the existing literature.^36,37^

The study’s strength is its comprehensive approach, employing a combination of IDIs and FGDs involving diverse key informant groups, thereby providing a nuanced comprehension of community attitudes towards mental illness. However, the purposive sampling of healthcare providers and community health workers could have resulted in an under-representation of alternative aetiologies and care modalities and an overemphasis on biological causes and biomedical interventions. The perspectives on supernatural causes of mental illness, particularly witchcraft, could reflect complex social dynamics in a post-colonial nation and within our research process.

While participants often characterised beliefs in witchcraft as outdated, published research on care-seeking pathways in Uganda shows that traditional healers remain frequently consulted for mental health concerns.^33^ This discrepancy may reflect our research team and positioning. The research assistants conducting the interviews were Ugandan master’s and bachelor’s students who were not local to the area. Their background was therefore a more urban demographic with more formal education than the study participants. Before starting the project, some initially expressed scepticism toward traditional healing practices, which could have influenced participant responses. The presence of US researchers during the interviews may have amplified participants’ perception of an external, potentially judgemental perspective, leading them to downplay traditional beliefs. This social positioning probably reinforced power dynamics where participants might have felt pressure to present perspectives aligned with biomedical frameworks. Stigma associated with TFHs, especially traditional healers, and influenced by a historical legacy of colonialism, might have contributed to social desirability bias, potentially leading to under-reporting of belief in such practices.^38,39^

Beyond these positioning considerations, our methodological approach presents additional limitations. Our decision to explore multiple mental health conditions (depression, anxiety, alcohol use disorder and suicide) within single focus group sessions was aimed at understanding how different conditions were conceptualised within the same cultural context. However, this approach may have limited the depth of discussion for each condition. The similarities in themes across conditions could partially reflect this methodological constraint rather than true uniformity in how these conditions are understood. Important distinctions may exist in how different mental health conditions are conceptualised and treated. Additionally, our study is limited by lack of data on participant sociodemographic characteristics, which could have provided important context for interpreting our findings and understanding how factors such as age, education and years of experience might influence perspectives on mental illness. Future studies should include such data to better understand how individual characteristics influence community beliefs about mental illness.

In conclusion, this qualitative study from eastern Uganda elucidated beliefs surrounding mental illnesses, encompassing psychosocial, biological and supernatural dimensions and treatments. Our findings accentuate the significant impact of poverty, particularly financial stressors, on mental health in the region. Community dynamics, as evidenced by the emphasis on community support, play a pivotal role in addressing mental health challenges. Given the central role of poverty, effective mental health interventions could also consider broader structural determinants, including food insecurity, economic opportunity and social protection systems. Because interventions tailored to local beliefs are crucial, the study lays the groundwork for targeted strategies that bridge conventional mental health services with community-centric care.

## Data Availability

The data that support the findings of this study are available from the corresponding author upon reasonable request.
